# Human amyloid-beta induces abnormal embryonic development and high embryonic lethality in *Caenorhabditis elegans*

**DOI:** 10.1093/bib/bbaf631.020

**Published:** 2025-12-12

**Authors:** King Law, Mao Onishi, Mitsuki Ohara, Yukihiko Kubota, Masahiro Ito

**Affiliations:** Graduate School of Life Sciences, Department of Bioinformatics, Ritsumeikan University; Graduate School of Advanced Studies, Laboratory of Genome Informatics, National Institute for Basic Biology; Graduate School of Life Sciences, Department of Bioinformatics, Ritsumeikan University; Graduate School of Life Sciences, Department of Bioinformatics, Ritsumeikan University; Graduate School of Life Sciences, Department of Bioinformatics, Ritsumeikan University

## Abstract

The aggregation of amyloid-β (Aβ) proteins is widely recognized as a key pathological biomarker of Alzheimer’s Disease (AD); its physiological function, however, remains largely unknown. Previous research has suggested that Aβ may regulate human brain embryogenesis and embryonic development in zebrafish. In this study, we used a *Caenorhabditis elegans* model of AD to explore the physiological function of human Aβ during embryogenesis. Analysis of embryonic lethality using strains CL2120 (human Aβ expressing) and CL2122 (empty vector control) shows a high embryonic lethality rate (31%), mostly occurring between the end of gastrulation to the 1.5-fold stage. RNAi screening and transcriptome analysis revealed that a key HOX developmental gene, *nob-1*, and its upstream regulator, *elt-1*, are significantly overexpressed in CL2120. As *nob-1* is heavily involved in posterior development at the end of gastrulation, our findings suggest that the elevated levels of ELT-1, induced by human Aβ expression, contribute to the observed high embryonic lethality. Taken together, our results indicate that Aβ proteins influence embryonic development by dysregulating key HOX genes.

